# Innate and Introduced Resistance Traits in Genetically Modified Aspen Trees and Their Effect on Leaf Beetle Feeding

**DOI:** 10.1371/journal.pone.0073819

**Published:** 2013-09-10

**Authors:** Joakim Hjältén, E. Petter Axelsson, Riitta Julkunen-Tiitto, Anders Wennström, Gilles Pilate

**Affiliations:** 1 Department of Wildlife, Fish, and Environmental Studies, Swedish University of Agricultural Science, Umeå, Sweden; 2 Department of Biology, University of Eastern Finland, Joensuu, Finland; 3 Department of Ecology and Environmental Science, Umeå University, Umeå, Sweden; 4 INRA, UR0588 Amélioration, Génétique et Physiologie Forestières, Orléans, France; Brigham Young University, United States of America

## Abstract

Genetic modifications of trees may provide many benefits, e.g. increase production, and mitigate climate change and herbivore impacts on forests. However, genetic modifications sometimes result in unintended effects on innate traits involved in plant-herbivore interactions. The importance of intentional changes in plant defence relative to unintentional changes and the natural variation among clones used in forestry has not been evaluated. By a combination of biochemical measurements and bioassays we investigated if insect feeding on GM aspens is more affected by intentional (induction Bt toxins) than of unintentional, non-target changes or clonal differences in innate plant defence. We used two hybrid wildtype clones (*Populus tremula x P. tremuloides* and *Populus tremula x P. alba*) of aspen that have been genetically modified for 1) insect resistance (two Bt lines) or 2) reduced lignin properties (two lines COMT and CAD), respectively. Our measurements of biochemical properties suggest that unintended changes by GM modifications (occurring due to events in the transformation process) in innate plant defence (phenolic compounds) were generally smaller but fundamentally different than differences seen among different wildtype clones (e.g. quantitative and qualitative, respectively). However, neither clonal differences between the two wildtype clones nor unintended changes in phytochemistry influenced consumption by the leaf beetle (*Phratora vitellinae*). By contrast, Bt induction had a strong direct intended effect as well as a post experiment effect on leaf beetle consumption. The latter suggested lasting reduction of beetle fitness following Bt exposure that is likely due to intestinal damage suffered by the initial Bt exposure. We conclude that Bt induction clearly have intended effects on a target species. Furthermore, the effect of unintended changes in innate plant defence traits, when they occur, are context dependent and have in comparison to Bt induction probably less pronounced effect on targeted herbivores.

## Introduction

Future forestry is expected to provide greater yields as well as environmentally cleaner products. This includes not only the traditionally important forestry product (e.g. timber and paper), but forests are also seen as an important tool for mitigating the predicted changes in climate [1]. Fossil fuels will need to be replaced with renewable energy sources and forests can, in theory, become a major source of bioenergy and thus reduce the anticipated rise in CO_2_ over the next 50 years. One way forward may be improvements in tree characteristics [1], [2] through genetic modifications.

Genetic modifications may help overcome some of the problems associated with conventional tree breeding. These problems include late flowering, slow maturation, long reproductive cycles, and complex mating systems (including self-incompatibility and a high degree of heterozygosity) in trees. Difficulties in identifying the best parents (and controlling their mating), maintaining genetic gain with high heterozygosity [3], and understanding the complex genome of many tree species also cause problems for tree breeders. Genetic modification (GM), on the other hand, theoretically allows modification of most individual traits in selected genotypes and is not hampered by slow maturation of trees. As a result, GM technology is much more specific than classical breeding and it can accelerate and allow new strategies for breeding [4].

Through genetic engineering it is possible to introduce novel traits as well as regulate native traits and thus change plant expression of various biochemical properties. The former type is most commonly used in transformations for pest resistance involving the use of *Bacillus thuringiensis* (Bt) genes, enabling the plant to produce Cry toxins lethal to certain targeted insect pests. More than 150 different Cry proteins have been identified [5]. For example, Cry3Aa proteins provide resistance to coleopteran insects and the cry1 and cry2 families effective against lepidopteran species [6], [7]. The effectiveness of these toxins against specific pest species of trees has been shown in the laboratory [8]–[10] and in the field [6], [11]. Other promising GM applications includes transformations to reduce lignin content in wood [12], [13]that could reduce the use of harmful chemicals and reduce energy consumption used in the de-lignification process in the pulp industry [13]. The decrease in lignin by bio-manipulation is occasionally associated with an increase in cellulose, thus further reducing the lignin to cellulose ratio, further highlighting the potential for an application within the pulp industry [14].

However, along with the beneficial effects many events in the transformation process may cause variability in gene expression or gene silencing and have secondary, unintended effects on plant physiology and fitness [15]–[17]. Some of these effects have been shown to affect traits involved in plant-herbivore interactions and decompositions processes, e.g. plant secondary metabolites [18], [19]. These changes in plant traits could potentially influence both benefits and ecological risks associated with GM trees and stresses the need for product-by-product evaluation to evaluate the potential benefits and risks with GM plants [19], [20]. These unintentional changes of innate plant defence sometimes influence e.g. plant-herbivore interactions. However, a question that remains to be addressed is the relative importance of these unintentional changes for plant herbivore interactions. How important are these unintentional changes compared to intentional changes in resistance traits, i.e. Bt induction, or the natural variation in resistance traits between different tree clones? To compare these effects is essential to our understanding of the relative impacts of GMOs on target and non-target species. We used two aspen hybrids (*Populus tremula x P. tremuloides* and *Populus tremula x P. alba*) genetically modified for induction of Bt toxin (providing insect resistance) and altered lignin properties (providing better pulping performance), respectively. The aim of this study was to test for differences in resistance traits and leaf beetle feeding between a) isogenic wildtype clones and the associated GM lines or b) between the two wildtype clones. More specifically, the following hypotheses were tested:

1. Genetic modification, i.e. induction of Bt toxins and changes in lignin characteristics of aspen, will result in significant (unintentional) changes in innate plant resistance traits (e.g. plant phenolics) as compared with unmodified isogenic wildtype clones.
2. Innate plant resistant traits will differ between the two wildtype clones and effect leaf beetle feeding.
3. Intentional induction of Bt toxins will have a stronger impact on *Phratora vitellinae* feeding than unintentional changes (resulting from GM modification process) or variation between wildtype clones in innate resistance traits (plant phenolics).
4. After exposure to leaves from GM lines, the negative effects on feeding will remain even when fed, non-GM tissue.

## Methods

### Plant Material

The wildtype (hereafter Wt) clones used were two aspen hybrids (*Populus tremula x P. tremuloides* and *Populus tremula x P. alba*) which have been genetically modified for insect resistance (Bt induction) [10] and altered lignin properties [13], respectively. Each of the two Wt clones (hereafter: Wt-Bt and Wt-Lignin) was contrasted in the experiments with two genetically modified lines, Bt17 and Bt27 as derived from the Wt-Bt clone, and CAD and COMT as derived from the Wt-lignin clone. Thus, the experimental setup consisted of a total of six aspen lines (two Bt lines (Bt17 and Bt27) with one control (Wt-Bt), and two lignin lines (CAD and COMT) with one control (Wt-lignin) for a total of four genetically modified lines and two Wt controls). This enabled comparisons between the two Wt clones as well as among the two Wt clones and their corresponding GM lines. The GM lines used in this experiment have been selected from a larger sample of GM lines due to their good performance in the lab and in the field [10], [13].

The two genetically modified Bt lines (Bt17 and Bt27) were previously described by [10]. They are modified to express a *cry*3Aa Bt-protein targeting coleopteran species. Bt17 and Bt27 produce toxins in concentrations of approximately 0.05% and 0.0025% of total soluble proteins in the leaves, respectively. Both lines have shown high resistance to the leaf beetle *Chrysomela tremulae* [10]. We also used the lignin modified lines CAD and COMT (referred to as ASCAD21 and ASOMT2B in [13]) that are modified to suppress cinnamyl alcohol dehydrogenase and caffeate/5-hydroxyferulate O-methyltransferase, respectively. Suppression of CAD leads to slightly decreased lignin content and modified structures in the lignin polymer resulting in wood with improved pulping characteristics. COMT is involved in the Syringyl lignin synthesis and suppression result in wood with poor pulping characteristics [13]. Along with the intended chemical changes in the wood, genetic modifications for altered lignin characteristics may also influence other properties of the plant such as the concentration of secondary substances expressed in the dormant leaves [18].

Plantlets of all lines were propagated in the lab and subsequently planted in 3 L pots in commercially available soil in the green house. The plants were distributed in a randomized block design with 7 blocks, each block consisting of one plant from each of the six lines, summing up to a total of 42 plants for the whole experiment. During the first 10 days of the establishment phase the plants were covered by individual micro-greenhouses using transparent plastic bags. After removal of the micro-greenhouses, the plants were left an additional 4 weeks before the feeding experiments started, thus they were 5 weeks old at the start of the experiment (i.e. when leaves were collected). Throughout the experiment standardized water and nutrient levels was provided to the plant through an automatic irrigation system. Supplementary light was applied throughout the experiment providing 16 h day length.

### No Choice Test and Post Experiment Performance

Adult *Phratora vitellinae* (Coleoptera; Chrysomelidae) individuals were collected in the field and to minimize the variation in plant responses due to variations in beetle life history state (e.g. sex and age) the beetles were randomly assigned to different treatments. Furthermore, the beetles were collected from the same site at the same time (i.e. they belonged to the same generation). This beetle species is a common herbivore on both willow and aspen species [21] and can be referred to as `salicyl-tolerant` as it converts salicyl glucosides from the host plant into a larval defensive secretion which consists mainly of salicylaldehyde [22], [23]. The leaf beetle used in the experiment *Phratora vitellinae* is a very common species that are regarded as a pest species in salix and aspen plantations. Thus, the species is not protected and no permit is required to collect this species in Sweden. Collection permits are only required for a small number of insect species in Sweden (http://www.naturvardsverket.se/upload/handbok/Bilaga%201_Svenska_djurarter_enligt_bilaga_4_habitatdirektivet.pdf). The beetle individuals were collected on land belonging to the City of Umeå and not on private or protected land and we therefore did not need any permits for our collections.

For the no choice test, one leaf was collected from a standardised position, leaf number 9 starting from the top of the plants with the first fully expanded leaf, from each of the plants. From each of the leaves two leaf discs were cut with a 13 mm Ø core borer. Each leaf disk was presented to one beetle in an experiment arena made up of one 100 mm Ø Petri dish with a moist filter paper attached in the top lid. In total 84 beetles were used in the tests. The beetles were left to feed in these arenas for 24 h after which the area eaten from each leaf disc was estimated with a 1-mm^2^ mesh size plastic screen. Reference discs (n = 7) from each line were also prepared, dried to constant weights (50°C) to establish specific leaf area ratio (SLA), i.e. leaf area to biomass ratio. The ratio of lignin and Bt lines differed significantly (P<0.001) but was the same within each group (P = 0.660 and P = 0.681 for Bt and lignin lines, respectively). Thus one SLA index was used for lignin and one for the Bt lines. These indexes were later used to convert consumed area to biomass.

Post-experiment performance (initiated the day after the no choice experiment was terminated) of the 84 *P. vitellinae* individuals used in the no-choice test was assessed by feeding them on native *P. tremula* leaf discs collected in the field. Biomass consumption was noted over a period of three days.

### Plant Chemistry

Leaves for chemical analyses were collected simultaneously as the leaves for the no-choice test but in this case leaf number 10 from the top was collected. All leaves were air-dried for 2 weeks at ∼22°C and milled before the analyses. To quantify the concentrations of individual secondary compounds, we used high-performance liquid chromatography (HPLC) for low molecular weight phenolics. Agilent’s Series1100 high-pressure liquid chromatography (HPLC) system (Agilent Technologies, Germany) equipped with Agilent’s G1315B diode array detector (DAD), and a reversed-phase (RP) octadecyl carbon chain (C18) column (Agilent Technologies, USA) was used. Leaf material (5 mg) was homogenized in 0.6 ml of methanol for 30 sec with an Ultra-Turrax homogenizer. The samples were then left in an ice bath for 15 min and then re-homogenized and centrifuged at 16 000 g for 3 min. The supernatant was collected, while the residue was washed three more times with 0.6 ml of methanol, homogenized for 30 sec and centrifuged. All supernatants were combined, and methanol was evaporated off in a vacuum centrifuge. The dried samples were dissolved in 150 µl of methanol and 150 µl of MilliQ water and were analysed for low molecular weight phenolics using HPLC. The compounds were separated on a 60-mm 4.6-mm column (HP Hypersil ODS II, 3 µm). The elution solvents were aqueous 1.5% tetrahydrofuran plus 0.25% orthophosphoric acid (A) and methanol (B). The gradient used have previously been described by [24]. The flow rate was 2 ml/min and the injection volume 20 µl. Individual compounds were identified by comparing their UV–visible spectra and retention time to those of known compounds. The quantification of salicin, chlorogenic acid, hyperin, kaemferol 3-glucoside, apigenin and tremulacin were based on commercial standards. The quantification of salicortin and tremuloidin were based on purified compounds from the leaves of *Salix* sp. The quantification of other compounds was based as follows: neochlorgenic acid based on chlorogenic acid; quercetins based on hyperin; isorhamnetin glycoside based on isorhamnetin 3-glucoside; apigenin derivative based on apigenin; monocoumaroyl-astragalin derivatives based on kaempferol 3-glucoside; HCH-tremulacin and tremulacin derivatives based on tremulacin. Soluble polymeric condensed tannins were measured from HPLC-samples with the butanol–HCl assay [25].

### Statistical Analyses

Phytochemistry profiles of leaves were explored with PERMANOVAs [26] using PRIMER (PRIMER-E, 2007) testing for the effect of the factor line. In these analyses we used Bray-Curtis distances in distance matrix construction and 4999 permutations. In case that the line factor indicated a significant effect, subsequent A-priori testing was conducted in accordance with our hypotheses (i.e. we tested for differences between the Wt lines and the associated modified lines or between the two Wt clones). Chemical profiles of leaves from the different lines and leaf positions were visualized with MDS plot, using Bray-Curtis dissimilarities. To clarify which substances contributed most to the observed differences in chemistry, we used Similarity Percentage Analysis (SIMPER), also on fourth-root transformed data. This is not a test of statistical probabilities *per se*, but a way of conceptualizing what differs between two sets of data: SIMPER calculates the overall percentage contribution that each substance makes to the average dissimilarity between two groups and lists the substances in decreasing order of their importance in discriminating between the two sets of samples [27].

Prior to analyses of the no choice test, the results for the two leaf discs collected from the same leaf were pooled. One way analyses of variance (ANOVA) were used to test for differences between the lines in the amount of leaf area consumed by the beetles in the no-choice and post experiment tests. A-priori testing was conducted in accordance with our questions (i.e. we tested for differences between the isogenic Wt clones and the associated modified lines or between the two wildtypes). In cases when the line factor indicated a significant effect subsequent pair-wise test were conducted using Tukey HSD test. The data for the no-choice test were Log [x+1] transformed to meet the assumption of homogeneity and normality. All statistical analyses were done with the statistical software SYSTAT 13 (Systat Software Inc. 2009).

## Results

### Phytochemistry

**Figure 1:**
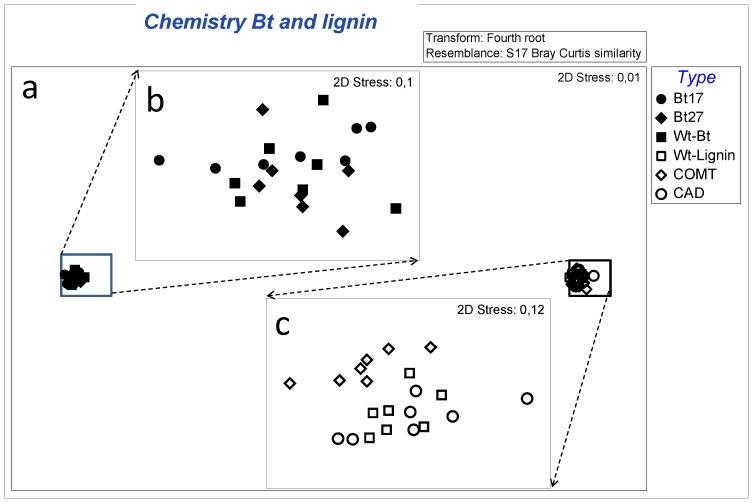
MDS plot illustrating the differences in chemical profiles among lines. The figures shows bigger variation in secondary chemistry differences between Wt clones (Figure a) compared to the differences between a specific Wt-lines and the associated GM varieties (Figure b and c, which is a magnification of figure a).

We only found partial support for our first hypothesis that phenolic substances should differ between the Wt and modified lines. Significant differences were found among the lignin lines (including CAD, COMT and Wt-lignin) (PERMANOVA Pseudo-F = 3.375, P = 0.002; Figure 1, Table 1). Line COMT had a significant different chemical composition compared to Wt-lignin and line CAD (P = 0.001 and P = 0.0018, respectively) but the latter lines did not differ significantly (P = 0.467; Figure 1). The SIMPER analyses show that these differences were without exception due to quantitative differences in secondary chemistry. For example, the COMT line contained higher concentration of quercetin diglycoside than the other lignin lines (Table 1). By contrast the secondary chemistry did not differ between the Bt lines (Bt17, Bt27 and Wt-Bt) (PERMANOVA Pseudo-F = 0.884, P = 0.504; Figure 1).

**Table 1:** Mean (+SE) concentration of phenolic compounds in the different plant lines and summary of the results from the SIMPER analyses.

Substance	Mean concentration (mg/g+SE)						COMT/Wt-lignin comparison		COMT/CAD comparison		Mean concentration (mg/g+SE)						Wt-lignin/Wt-Bt comparison	
	Wt-lignin	SE	CAD	SE	COMT	SE	Contr %	Direction	Contr%	Direction	Wt-Bt	SE	Bt17	SE	Bt27	SE	Contr %	Direction
Quercetin diglycoside 1	0,76	0,11	0,54	0,10	1,69	0,18	9,20	(+)	10,05	(+)	0,61	0,10	0,54	0,09	0,49	0,05	1,02	(+)
Quercetin glycoside der 1	0,73	0,09	0,49	0,06	1,89	0,08	8,35	(+)	9,76	(+)	0,00	0,00	0,00	0,00	0,00	0,00	9,71	(+)
Quercetin-diglycoside 2	0,09	0,01	0,09	0,02	0,26	0,07	7,09	(+)	8,63	(+)	0,60	0,10	0,63	0,14	0,58	0,05		
Quercetin diglycoside 3	0,09	0,01	0,06	0,01	0,29	0,03	6,75	(+)	6,80	(+)	0,44	0,06	0,43	0,08	0,41	0,03		
Rhamnetin der 1	2,68	0,30	2,90	0,25	1,53	0,17	5,04	(−)	4,92	(−)	0,00	0,00	0,00	0,00	0,00	0,00	10,54	(+)
Kaempferol glycoside1	0,23	0,03	0,17	0,03	0,14	0,02	4,66	(−)	3,25	(−)	0,14	0,01	0,11	0,01	0,12	0,01		
Hyperin	5,16	0,65	5,78	0,95	4,36	0,59	3,71	(−)	3,83	(−)	0,00	0,00	0,00	0,00	0,00	0,00	10,97	(+)
P-OH-cinnamic acid der	1,05	0,08	1,12	0,09	1,17	0,10	3,57	(+)	3,89	(+)	2,75	0,19	2,99	0,38	2,73	0,19		
Chlorogenic acid	2,77	0,42	2,88	0,51	2,91	0,34	3,32	(+)	2,81	(+)	0,00	0,00	0,00	0,00	0,00	0,00	10,54	(+)
Monocoumaroyl astragalin2	1,07	0,10	1,08	0,15	1,03	0,15	3,25	(−)	3,15	(−)	0,45	0,07	0,31	0,04	0,50	0,13		
Rhamnetin der.2	0,41	0,04	0,50	0,05	0,57	0,06	3,20	(+)	2,20	(+)	0,00	0,00	0,00	0,00	0,00	0,00	9,37	(+)
HCH-tremulacin3	1,64	0,19	1,49	0,16	1,41	0,15	3,15	(−)	2,36	(−)	3,27	0,19	3,11	0,19	3,06	0,08		
Monocoumaroyl astragalin1	0,53	0,05	0,57	0,08	0,50	0,07	2,97	(−)	3,13	(−)	0,13	0,02	0,09	0,01	0,16	0,04		
Salicortin	67,68	7,33	57,90	6,13	66,99	4,65	2,90	(−)	2,70	(+)	144,25	3,72	150,57	4,58	153,59	5,65		
Pinocembrin1	0,17	0,02	0,15	0,02	0,17	0,02	2,82	(−)	2,46	(+)	0,09	0,00	0,10	0,01	0,10	0,01		
Tannins	3,18	0,16	3,82	0,60	4,18	1,11	2,73	(+)	3,14	(+)	3,64	0,18	4,09	0,27	3,85	0,17		
HCH-salicortin	3,27	0,33	2,87	0,30	2,80	0,22	2,64	(−)	2,15	(−)	4,60	0,31	4,71	0,22	5,12	0,26		
Disalicortin	3,20	0,29	2,92	0,26	2,86	0,26	2,38	(−)	2,03	(−)	9,90	0,77	10,45	0,69	10,25	0,45		
Tremulacin	94,08	6,60	81,39	8,24	88,62	5,16	2,15	(−)	2,36	(+)	129,58	6,53	124,61	9,60	126,01	7,81		
Pinocembrin2	0,13	0,01	0,11	0,01	0,13	0,01	2,14	(+)			0,00	0,00	0,00	0,00	0,00	0,00	8,70	(+)
Diglucoside of saoh	1,75	0,09	1,79	0,20	1,66	0,19	2,14	(−)	2,38	(−)	1,06	0,12	0,88	0,08	1,12	0,12		
Salicin	3,36	0,14	3,18	0,26	3,22	0,33	2,09	(−)	1,93	(+)	4,96	0,43	4,87	0,42	4,77	0,39		
P-OH-cinnamic acid der	4,40	0,57	3,93	0,80	4,34	0,58	2,06	(−)			2,21	0,19	2,77	0,33	2,54	0,15		
Chlorogenic acid der 1	0,24	0,02	0,22	0,02	0,23	0,02	2,05	(−)	3,50	(+)	0,34	0,01	0,29	0,01	0,36	0,01		
(Neo)chlorogenic acid der	4,31	0,26	4,31	0,41	4,56	0,44			2,03	(+)	6,18	0,36	5,54	0,36	6,24	0,25		
HCH-tremulacin1	0,96	0,09	0,84	0,08	0,76	0,03			1,86	(−)	1,93	0,07	1,81	0,11	1,91	0,07		
Hyperin+kaempferol glycoside	0,00	0,00	0,00	0,00	0,00	0,00					5,53	0,81	5,66	0,83	5,03	0,43	11,00	(−)
Chlorogenic acid der 2	0,51	0,04	0,46	0,08	0,56	0,05					0,00	0,00	0,00	0,00	0,00	0,00	9,71	(+)
Kaempferol glycoside2	0,00	0,00	0,00	0,00	0,00	0,00					0,50	0,03	0,49	0,03	0,49	0,02	9,50	(−)
kaempferol 3-rhamnoside	5,74	0,22	5,84	0,32	5,08	0,33					9,60	0,85	9,72	0,79	9,50	0,52		
HCH-tremulacin2	1,02	0,09	0,90	0,08	0,83	0,04					2,01	0,08	1,91	0,10	1,95	0,04		

Percentage contribution of different substances to the total dissimilarity is given for the significant comparisons found in the PERMANOVA analyses. Direction indicates if the mean value is higher or lower in the first line in the comparison.

Consistent with our second hypothesis, secondary chemistry differed between the two non-modified Wt lines (Wt- Bt and Wt-Lignin). In fact, the largest differences in secondary chemistry in this study was found in comparisons of Wt-Bt and Wt-Lignin (PERMANOVA Pseudo-F = 260, P = 0.0002; Figure 1, Table 1, also indicated by the extremely low stress level of 0.01 in the nMDS plots). Some of these differences are clearly attributed to qualitative differences in secondary chemistry between Wt-Bt and Wt-Lignin (Table 1). For example, the SIMPER analyses revealed that several compounds that occurred, sometimes in high concentrations in WT-Lignin (e.g. hyperin and chlorogenic acid) were missing in Wt-Bt and vice versa. For example, 9 of the 31 compounds detected were unique to one of the Wt lines (Table 1).

### Herbivore Assays

**Figure 2:**
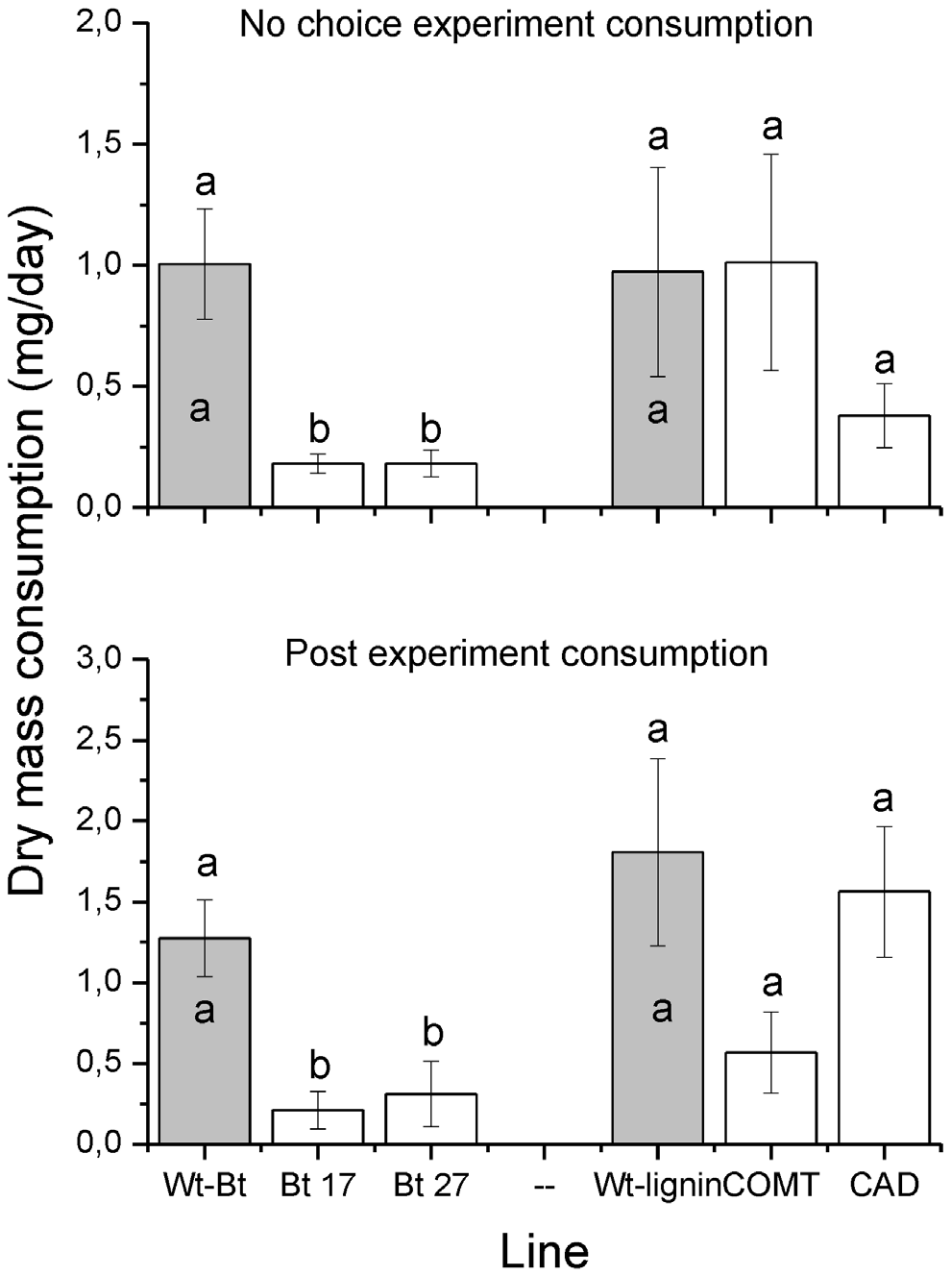
Results from the no-choice and post experiment (feeding on *P. tremula*) feeding trials. Bars shows the mean consumed dry mass per day from the two Wt hybrids (Wt-Bt and Wt-Lignin,) and their genetically modified varieties (Bt17 and Bt27, and CAD and COMT, respectively). Different letters above bars denotes significant differences between a specific Wt line and their associated GM varieties. Different letters inside bars denotes significant differences between the two Wt lines (Tukey test P<0.05).

We found support for our third hypothesis that Bt induction is expected to have a stronger effect on leaf beetle feeding than unintentional changes in plant phenolics. Consumption by *P. vitellinae* differed between Wt-Bt and the Bt lines (ANOVA F = 7.240, P = 0.005). Both the Bt lines were consumed significantly less than Wt-Bt (Figure 2). No significant difference was found between the Bt lines (Figure 2). No differences were found between the modified lignin lines and Wt-Lignin (ANOVA F = 0.227, P = 0.799, Figure 2). Consumption did not differ significantly between Wt-Bt and Wt-Lignin (ANOVA F = 0.13, P = 0.358, Figure 2).

In support of our fourth hypothesis, the post experiment consumption, during which the beetles used in the experiments were offered leaves from naturally growing native aspen, showed differences between the beetles previously feeding on the Bt lines and Wt-Bt (ANOVA F = 9.371, P = 0.002). The beetles that had experienced feeding from either of the two Bt expressing lines during the experiment consumed less leaf mass than beetles that consumed Wt-Bt leaves (Figure 2). No difference in consumption was found between beetles that had consumed leaves from the lignin lines and Wt-Lignin during the experiment (ANOVA F = 2,298, P = 0.129, Figure 2). In addition, no difference in post experiment consumption was found between Wt-Bt and Wt-Lignin (ANOVA F = 0.724, P = 0.411, Figure 2).

## Discussion

### Unintentional Effects

In opposition to our first hypothesis, we found no differences in secondary chemistry between the Bt lines and Wt-Bt. This contradicts earlier findings of differences in secondary chemistry between these lines [28]. However, in [28] the differences in chemistry coincided with clear differences in growth between Wt-Bt and modified Bt lines. The differences in growth were less pronounced in another study [29] and in the present study reported here. Growth may affect phytochemistry of poplar trees [30] and plant vigor may indeed influence resistance properties [31], [32]. One additional factor that potentially could account for the discrepancy between this and earlier studies is that the leaves used in this study were phenologically older, 10 leaf from the top compared to 3 or 6 leaf from the top in [28]. The differences seen in both growth and expression of phytochemistry among studies using the same lines suggest growth dependent conditionality that should be explored in future studies. A compelling consequence of the lack of differences in secondary chemistry between the Bt-lines (Wt-Bt, Bt17 and Bt27) is that any observed differences in leaf damage by *P. vitellinae* on the Bt-lines is likely to be an effect Bt expression in the leaves and not due to any unintended changes in innate defence (i.e. phenolics) in modified lines. This strengthens earlier reports of Bt effectivity in these lines [9]–[11].

Furthermore, we found no significant effect of genetic modification on the palatability on the lignin modified lines, despite the fact that we found significant differences in secondary chemistry between line COMT and both the Wt-lignin and CAD lines, e.g. the concentration of the four quercetin diglycoside derivates were twice as high in the COMT line than in the CAD and WT-lignin lines. By contrast, the concentration of Rhamnetin was ca 50% lower in the COMT line than in the CAD and WT-lignin lines. Earlier studies have reported unintentional changes in plant palatability in GM trees, possibly related to observed changes in plant chemistry [19], [20], [28]. One possible explanation for the lack of a beetle response to chemical variation in our study is that the induced changes in innate resistance traits was best explained (32–35% of the variation explained, Table 1) by higher concentrations of phenolic glucosides (quercetin diglycoside derivates) in COMT compared to the Wt-Lignin and CAD lines. *P. vitellinae* uses phenolic glucosides to synthesis their own defence compounds [22], [33], [34]. Thus, induced levels of one important group of phenolic glucosides (salicylates) have shown little negative effect on feeding by the highly specialized *P. vitellinae* [34]–[36]. In contrast, they appear to avoid feeding on willow species with high tannin concentrations [33] and larvae of *P. vitellinae* grow less well on willows with high concentration of condensed tannins [22], [37], [38]. However, concentration of tannins was similar in the different lignin lines (4.2, 3.8 and 3.2% for COMT, CAD and Wt-Lignin, respectively) and differences in tannin concentration only explained 2.7% of the variation in secondary chemistry between lignin lines (Table 1). Thus, these similarities in tannin correspond well with the lack of responses seen in consumption. In a study of another type of lignin modification in birch, no effects on feeding by five associated insect herbivores were detected [39]. By contrast, [19] reported a significant effect of genetic modification on *P. vitellinae* preference, but in this case a significant effect on plant tannin concentration was also detected.

### Clone Differences

Our second hypothesis was only partially supported. We found very clear differences in secondary chemistry between the two non-modified WT clones, Wt-Bt and Wt-Lignin. The difference in secondary compounds was much larger between Wt clones than between GM lignin lines CAD and COMT and the Wt-Lignin, mainly due to qualitative differences in phenolic composition between the Wt clones. This is not surprising as the Wt-Bt is a *Populus tremula x P. tremuloides* hybrid whereas Wt-Lignin is a *Populus tremula x P. alba* hybrid. The parental species exhibit differences in their secondary chemistry [40], [41] which seem to be expressed also in the Wt-hybrids.

However, these differences in secondary chemistry between the Wt-clones did not translate into differences in utilization by *P. vitellinae*. Inter and intraspecific variation in *Populus* ssp traits can have a strong effect on herbivore growth and survival [42]–[45]. The reason for this lack of response is unclear but *P. vitellinae* has been found attracted by two related salicylate glucosides, tremulacin and salicortin [46] and none of these explained any significant part of the variation in secondary chemistry, only 0.65% in the latter case. In addition, tannin concentration, reported to have negative effect on *P. vitellinae* [22], [37], [38] had no influence at all on the difference in secondary chemistry between the Wt-clones. One likely reason for this is that young trees usually are high in glucosides and low in tannins [47], as was also the case in our study (Table 1). Thus, both these clones might have sufficient high quality for this specialist herbivore not to differentiate between them. [47] also found that specialist insect herbivores only were marginally affected by clone variation in secondary chemistry. However, other differences in plant chemistry (e.g. nitrogen, concentrations of specific tannins) and leaf structure (toughness, hairiness, fibre content) between the two Wt-clones could have influenced palatability and potentially counteracted quality differences resulting from differences in phenolic composition [48], [49]. For example, we found that the reference discs from the two wildtypes differed in SLA ratio which could indicate some structural differences between leaves. The SLA ratio of the lignin line is approximately 24% higher than in the Bt lines which coincide with the visual impression of the two lines in which the lignin line have more robust leaves that grow more vertical from the stem. Such leaf toughness` characteristics may have a strong influence over plant palatability towards insect herbivores [50]–[52].

### Relative Influence on Feeding

In accordance with our third hypothesis, intentional Bt induction had a stronger impact on *P. vitellinae* feeding than unintentional changes or clone variation in phytochemistry. Both Bt lines were less consumed than the Wt-Bt whereas Bt induction had no effect on secondary chemistry and lignin modification had no effect on feeding. This is consistent with earlier studies suggesting that Bt makes aspens more resistant to various insect pests [6], [9]–[11]. The fact that beetles consuming Bt leaves during the experiment, also showed post experiment reduction in leaf biomass consumption suggest, if not permanent, at least lasting reduction in feeding ability following Bt exposure. This could potentially be explained by the mode of action of Bt toxins which binds to receptors on cells lining in the larval midgut, inserts into the cell membrane, and forms ion channels resulting in loss of the transmembrane potential which leads to osmotic cell lysis [53]. At sub-lethal concentrations of toxin, there are larval behavioural changes such as avoidance of the toxin during feeding and paralysis of feeding [54], which could explain both the immediate reduction levels of leaf damage and the lasting effect of Bt exposure on feeding. Feeding on leaves from Bt aspen is deleterious for another leaf beetle (*Chrysomela tremulae*) regardless of the developmental stage [10] and also severely reduced reproduction of *P. vitellinae* [9].

### Contrasts of Different Genetic Modifications

One important objective with this study was to compare differences in secondary chemistry and its influence on palatability, between different aspen clones and among different genetic modifications. Our measurements of biochemical properties suggest that unintended changes in innate resistance traits were generally smaller than differences that can be seen among different aspen hybrids clones. Nevertheless, it is also shows that the natural differences in phenolic composition among clones were fundamentally different compared to differences due to genetic modifications; clone differences were predominantly qualitative, whereas unintended changes between Wt and their respective GM lines were exclusively quantitative. Nine of the ten substances that explained most of the difference in secondary chemistry between the Wt clones were completely missing from one of the Wt clones (Table 1). The relative ecological influences of these differences are hard to evaluate. Further investigation including other herbivore species is needed to determine if this lack of difference in palatability between the two Wt clones is general or species specific. Thus, in this study we are unable to determine if unintended changes in innate defence (secondary chemistry) resulting from genetic modifications has more or less influence on plant palatability to herbivores than differences due to natural variation among different clones of trees.

In summary, we found that targeted changes in aspen defence such as Bt toxin induction had strong effects on the feeding by a potential insect pest and that this also had post exposure effects on the insect. The non-target or unintentional changes in native plant chemistry following genetic modification were considerably lower but fundamentally different than naturally occurring differences between the wildtype hybrid clones. However, neither unintentional changes nor clone variation in plant secondary chemistry had any significant effects on insect feeding. Thus, we were unable to evaluate the relative importance of unintentional changes in plant chemistry in GM plants compared to naturally occurring clonal variation.
